# The Potential Relevance of the Microbiome to Hair Physiology and Regeneration: The Emerging Role of Metagenomics

**DOI:** 10.3390/biomedicines9030236

**Published:** 2021-02-26

**Authors:** Andria Constantinou, Varvara Kanti, Katarzyna Polak-Witka, Ulrike Blume-Peytavi, George M. Spyrou, Annika Vogt

**Affiliations:** 1 Charité-Universitatsmedizin Berlin, Corporate Member of Freie Universitaet Berlin and Humboldt-Universitaet zu Berlin, Clinical Research Center for Hair and Skin Science, Department of Dermatology, Venereology and Allergy, Charitéplatz 1, 10117 Berlin, Germany; Andria.constantinou@charite.de (A.C.); vera.kanti@charite.de (V.K.); katarzyna.polak-witka@charite.de (K.P.-W.); ulrike.blume-peytavi@charite.de (U.B.-P.); 2 Bioinformatics ERA Chair, The Cyprus Institute of Neurology and Genetics, 6 Iroon Avenue, 2371 Ayios Dometios, Nicosia, Cyprus; georges@cing.ac.cy

**Keywords:** follicular microbiome, hair follicle, hair disease, hair loss, alopecia, metagenomics, next generation sequencing, bioinformatics, bacteria, dysbiosis

## Abstract

Human skin and hair follicles are recognized sites of microbial colonization. These microbiota help regulate host immune mechanisms via an interplay between microbes and immune cells, influencing homeostasis and inflammation. Bacteria affect immune responses by controlling the local inflammatory milieu, the breakdown of which can result in chronic inflammatory disorders. Follicular microbiome shifts described in some inflammatory cutaneous diseases suggest a link between their development or perpetuation and dysbiosis. Though the hair follicle infundibulum is an area of intense immunological interactions, bulb and bulge regions represent immune-privileged niches. Immune privilege maintenance seems essential for hair growth and regeneration, as collapse and inflammation characterize inflammatory hair disorders like alopecia areata and primary cicatricial alopecia. Current research largely focuses on immunological aberrations. However, studies suggest that external stimuli and interactions across the follicular epithelium can have profound effects on the local immune system, homeostasis, and cycling. Herein, we review hair follicle bacterial colonization, its possible effects on the underlying tissue, and links to the pathogenesis of alopecia, beyond the pure investigation of specific species abundance. As skin microbiology enters the metagenomics era, multi-dimensional approaches will enable a new level of investigations on the effects of microorganisms and metabolism on host tissue.

## 1. Introduction

The surface of human skin is a site of microbial colonization [1,2]. The commensal flora of the skin maintain a symbiotic relationship and shape the cutaneous immunity of the host [3,4]. Several studies have characterized the cutaneous bacterial communities of healthy individuals and patients with inflammatory diseases, mostly in material collected using swabs [5,6,7,8,9]. Such sampling from hair follicle (HF) openings, however, is especially challenging. Along their epithelium, HFs harbor a multitude of microorganisms able to reach deeper compartments [10], frequently organized in biofilms [11,12]. As only few animal models allow to address scientific questions related to those events, hair research largely relies on patient samples, i.e., biopsies, though hair plucking is increasingly used [11,13]. While the role of bacteria as disease-aggravating factors has been recognized for HF-associated inflammatory diseases such as acne vulgaris or folliculitis decalvans [12,14,15], only scarce information is available on how changes in the follicular microbial communities can affect HF immunology, cycling, and regeneration.

## 2. Bacterial and Host Crosstalk

There is increasing proof of the presence of bacteria reaching below the skin surface and along the HFs [10,11,16], and rising evidence is emerging that the cross-talk between bacteria and the underlying tissue is a dynamic reciprocal process [17,18]. External microbial stimuli create immunological responses across the HF epithelium [19], but events in the tissue also shape microbial composition [12]. Along the infundibular HF epithelium, microorganisms find themselves in a highly immuno-active environment. Their presence triggers innate immune responses via the induction of antimicrobial peptides and cytokines [20,21], but this specific anatomic niche further provides access to antigen-presenting cells [22,23] and sets the ground for immunomodulatory interactions with wider implications for tissue homeostasis and regeneration.

HF morphogenesis, commensal microbe colonization, and local chemokine production work in concert to establish a regulatory T cell (T-regs) niche early in life [24]. Additionally, HFs appear to be crucial for tissue-immune crosstalk, but they depend on macrophage for quorum sensing induced regeneration [25], T-regs for HF stem cell differentiation [26], and innate lymphoid cells for sebaceous gland control [27]. Innate lymphoid cells in the vicinity of the sebaceous gland affect sebaceous gland function and microbial commensals by producing cytokines and growth factors that promote barrier function and the expulsion of pathogens [27].

## 3. Hair Follicle Immune Privilege and Disease

Anatomic sites of immune privilege (IP) can be found in the brain, eyes, gonads, and skin, and provide a microenvironment which restricts immune-mediated inflammation, e.g., by suppression of antigen-recognition pathways [28,29]. Various mechanisms determine IP including confluence of cells, cellular factors, architectural barriers, or the absence of lymphatic drainage [29]. However, IP development is a dynamic process that, in order to be sustained, often requires only a subset of these mechanisms, resulting in a “relative” IP status with a particular degree of immune protection [28].

As for the HF, the bulb and bulge region display a characteristic downregulation of major histocompatibility complex (MHC) class I and II expression [30,31]. As well as local expression of immunoregulatory and immunosuppressive factors, including proopiomelanocortin-derived neuropeptide (α-MSH), members of growth factor family (TGF-β1, IGF-1) and mediators like the macrophage migration inhibitory factor that actively suppresses NK cell responses [30,31]. Once established, IP remains a dynamic state [32]. Restoration of the bulb IP during the anagen phase is essential to maintain hair growth [33,34]. Impairment of IP maintenance has been associated with pathogenesis and perpetuation of inflammatory hair disorders [35]. Based on the marked peribulbar inflammatory infiltrate of anagen HFs in alopecia areata (AA), the collapse of the bulb IP is believed to be a central event in the inflammatory destruction and subsequent inability to regrow hair during active flare-ups [36,37].

Similar to the findings in the bulb, characteristics of a relative IP including decreased cell-surface markers and β-2-microglobulin, as well as increased CD200 (an immunoregulatory glycoprotein) were found in the bulge region [38,39], which harbors epithelial stem cells essential for HF regeneration. Based on those findings, the concept of IP breakdown was extended to primary cicatricial alopecias (PCA), e.g., lichen planopilaris, which primarily involves inflammatory infiltration and subsequent destruction of the bulge region [35,40,41]. However, it is unclear whether IP collapse occurs early in the disease process before inflammation develops or later as a secondary phenomenon [35].

## 4. Could External Factors Negatively Impact IP Maintenance?

Considering the multitude of microorganisms residing in follicular openings, the question arises whether external factors could impact the immunological activation state of the underlying tissue, thereby facilitating the disruption of the local IP. Anatomically, the infundibulum would be the most obvious site for such interactions. This rather superficial compartment has long been recognized as a site of intense immune cell trafficking and activation [42], but also pathogen invasion, as observed in ostiofolliculitis [43]. The barrier function of the epithelium is less developed in deeper parts, which increases the likelihood of tissue penetration for exogenous compounds, including larger molecules such as nucleic acids, proteins, particles, and viruses [44,45,46]. Penetration studies point towards an enhanced penetration and a special vulnerability of the HF during the anagen phase of the hair regrowth circle, a time when hair growth crucially depends on a robust immune privilege around the bulb [47]. Hence, epithelial compartments in the vicinity of the bulge could be possibly exposed to immunoregulatory and/or pro-inflammatory stimuli from the outside, but also the inside, as a result of cross-talk initiated by external factors.

A diversity of environmental, genetic, hormonal, or stress-related triggering factors have been attributed to the development of PCA [35,48,49,50]. The observation that lichenoid inflammation can be triggered is a well-known phenomenon (i.e., isomorphic response or *Koebner* phenomenon) [51]. However, the contribution of external disruptors of HF homeostasis to the local onset of inflammatory hair diseases has not been deeply studied, although this idea has gained attention due to the rising incidence of frontal fibrosing alopecia (FFA), long considered a lichenoid disease with a peculiar clinical pattern. Links to leave-on facial products, sunscreen use, allergens, and specific foods have been debated [52,53,54], but with no conclusive etiological connection to date [55,56,57].

## 5. What Do We Know about Bacteria in HF-Associated Disorders and Hair Diseases?

Regarding the contribution of bacteria to disease aggravation and progression, acne vulgaris is a well-studied HF-associated disease entity. Sanford et al. demonstrated how metabolic products of skin-resident bacteria (*Cutibacterium acnes*, *C. acnes*) in certain settings can induce inflammatory gene and cytokine expression from sebocytes to promote inflammatory acne [58]. Accordingly, upregulation of inducible antimicrobial peptides such as beta-defensins was demonstrated in lesional and perilesional epithelium of acne biopsies [59]. Interestingly, no differences in relative abundances of *C. acnes* in patients with acne and healthy subjects were observed, whereas strain population structures were significantly different among the groups [60]. While most studies focus on bacterial abundance, the presence of specific strains or even specific species co-existence might be of relevance.

Hidradenitis suppurativa (HS) is another HF-associated chronic inflammatory skin disease for which triggering microbial factors have been suggested [61,62,63]. Bacteria with biofilm formation capacities, especially coagulase-negative staphylococcus, trigger a cascade of pathogen-associated molecular patterns, leading to inflammasome activation and release of pro-inflammatory cytokines (e.g., interleukin-1β) regulating inflammation, tissue repair, and tissue death [64]. Recently, next-generation sequencing showed that a wider spectrum of species is involved [62]. HS could help us better understand other biofilm-driven diseases, like folliculitis decalvans. Based on the work of Ring et al., where less biofilm structures and less bacteria were found in unaffected axillary skin of HS patients compared to controls [65], we are challenged by homeostasis complexity and the risks of prejudicing high bacterial abundance as a disease causative.

The field is still in the early stages of building a more refined understanding of the role of bacteria in the different alopecia subtypes. Folliculitis decalvans (FD) is probably the best characterized scalp disease concerning bacterial contribution to HF inflammation. *Staphylococcus aureus* (*S. aureus*) was isolated from most untreated FD patients, and long-term remission after oral antibiotic treatment has been widely reported [9,66,67,68]. It is hypothesized that cytotoxic proteins secreted by *S. aureus* act as superantigens and stimulate T cells. In genetically predisposed individuals, innate immunity mechanisms (e.g., IL-8) induce an intense neutrophil migration in the peri- and intra-follicular dermis [69,70], resulting in damage of the follicular epithelium and subsequent release of proinflammatory (IFN-γ, TNF-α) and profibrotic mediators (TGF-β, b-FGF, IL-1β, IL-4) that activate fibroblasts [70]. Presence of *S. aureus* was found to be enhanced in scalp skin of FD patients and, interestingly, superficial presence in lesional skin increased the chance of lesional subepidermal colonization [71].

**Figure 1:**
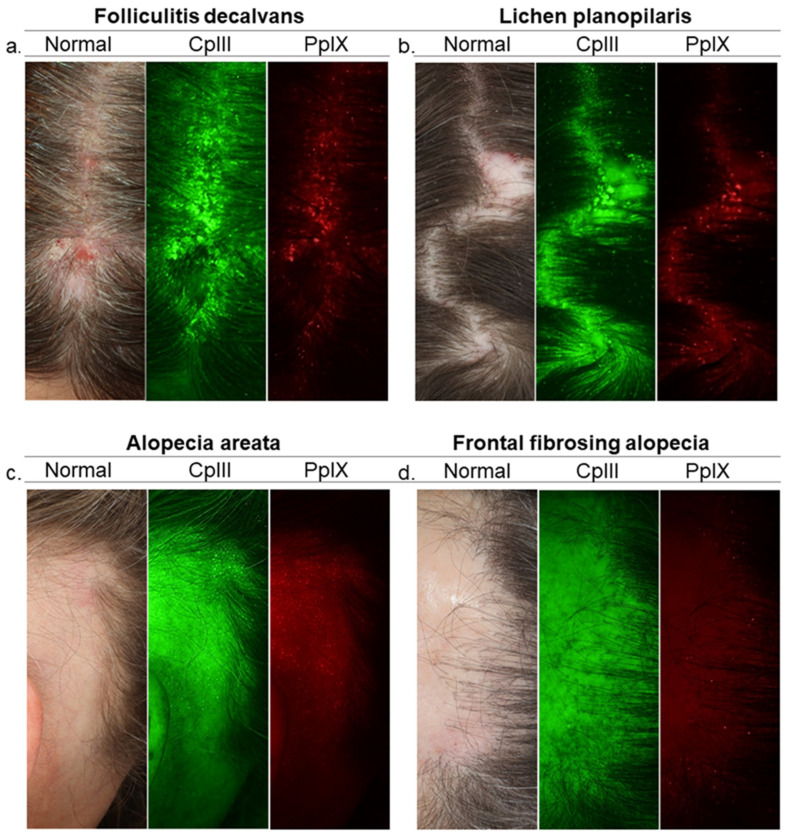
Need for a deeper understanding of HF microbiome. Images of Visia-CR Porphyrins Analysis in scalp diseases. Porphyrins, Cutibacterium acnes excretions, fluoresce in UV light and exhibit circular bright spot characteristics. Porphyrins are more numerous on the lesional scalp of folliculitis decalvans patients and scarce on frontal fibrosing alopecia patients. Yet, there is reason to believe that events of greater complexity which are crucial for HF health and disease take place deeper. Diving deeper into bacterial communities and interactions with the HF’s epithelial lining may help unlock the secrets of regulatory processes along the HF, revealing essential information for the quest of the missing links between follicular microbiome and disease. Images from left to right, Standard White Light Image (Normal Clinical Photograph); Coproporphyrin (CpIII) Fluorescence Image and Protoporphyrin (PpIX) Fluorescence Image of the scalp surface (follicular openings and the interfollicular area). Green and red fluorescence spots correspond to CpIII and PpIX respectively. HF, hair follicle.

However, studies show a variety of HF bacteria in FD patients including *C. acnes* in biofilm-like structures [12,72], implying the involvement of other bacterial species. Alternatively, it suggests the hypothesis of a follicular reservoir primarily consisting of *C. acnes*, whose organization in biofilms provides protection and stability, considering that *C. acnes* is also found in healthy subjects [11,71,73] (Figure 1). How the local microenvironment and signaling across the barrier triggers a shift from a state of immunological tolerance to a state of activation and chronic inflammation are complex.

A good example of how the local follicular microenvironment (e.g., hypoxic conditions, lipid substrates), resident microbes, and their metabolic products can influence inflammatory and antimicrobial processes in the skin is the aforementioned acne vulgaris [58]. Similarly, Folliculitis et perifolliculitis capitis abscedens et suffodiens, a rare chronic inflammatory scalp disease, is considered to be an inflammatory reaction to microbial components of the HF, particularly bacteria (especially *C. acnes* or *S. aureus*), yeasts (Malassezia species), and mites (Demodex folliculorum) under certain conditions. The main pathophysiology mechanisms involved are enhanced by an inflammatory bacterial process resulting in chemotaxis of neutrophils [74].

**Figure 2:**
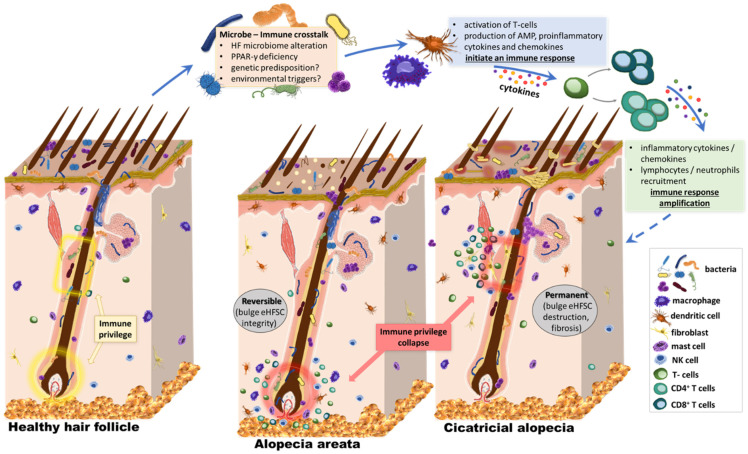
Our hypothesis. The effect of the microbiome on HF stem cell niche protection and survival. The skin surface and follicular openings are recognized sites of rich microbial colonization and intense immune activation, with cross-talk across the skin barrier. The bacterial microbiome extends below the infundibulum, with the confirmed presence of various species and high abundance of Corynebacterium and Staphylococcus. It is in close proximity to structures of the immune privileged status, which are essential for the hair cycle. Several preliminary studies suggest that external stimuli could affect the state of activation of the HF immune system. Lower sections of the HF are protected from immune cell infiltration under healthy conditions, the so-called “immune-privileged” areas. This includes the bulge, where a stem cell niche is found, and the bulb, where cells divide and grow to build the new hair. However, both regions are sites of intense inflammatory infiltrate in inflammatory hair diseases like primary cicatricial alopecia and alopecia areata. Alterations in the HF microbiome or the penetration depth of microbial material could be related to homeostasis, modulation of cutaneous immune reactions, and inflammatory processes along the HF. The localization of this involvement with the immune system is critical, affecting the possibility of regrowth after subsidence of inflammation. When the stem cell niche is attacked, like in cicatricial alopecia, patients suffer permanent, scarring hair loss. On the other hand, AA pathogenesis involves inflammation of the peribulbar region, allowing a possible hair regrowth. The role of microbiota in the pathogenesis of alopecia is proposed, but in ways more complex than just bacterial abundance of specific species in the area. HF, hair follicle.

Nevertheless, could other PCA unveil an underlying infection or follicular microbiome alterations? How the HF microbiome possibly preserves IP, and how shifts affect the state of activation and regeneration needs to be explored. Herein, we hypothesize that a dynamic change in the scalp follicular constituents triggers a distinct transcriptional response, alters the local immune system regulations, and activates immune cells, resulting in inflammatory responses, IP collapse, and pathologies like PCA or AA (Figure 2). Whether HF’s regenerative ability is preserved depends on the immune attack localization and the survival of HF stem cells. Microbiome shifts in deeper follicular compartments may attract dense immune peribulbar infiltration (e.g., NK cells observed in AA), potentially causing hair loss but maintaining hair regrowth capacity after inflammation regresses. Our hypothesis is that in PCAs, like lichen planopilaris or FFA, a superficial change in the composition of follicular microbiota could cause a cascade of pro- and inflammatory chemokines around the bulge. This cascade may activate immunological pathways that attract immune cells around this sensitive area, ultimately causing scarring and permanent hair loss.

Dandruff is associated with bacterial and fungal scalp populations [75,76], while recent studies are also focusing on androgenetic alopecia and AA. Micro-inflammation in androgenetic alopecia consists of upper perifollicular infiltrate, suggesting a triggering effect near the infundibulum [77]. A reported improvement after using an antimicrobial lotion indicates the involvement of scalp’s common inhabitants [78]. A recent study by Ho et al. shows alterations in the microbiota of the middle and lower compartments of miniaturized HFs, specifically an increased abundance of *C. acnes* [79]. Additionally, Malassezia have been shown to have a positive correlation with the incidence of androgenic alopecia [80]. Pinto et al. found a significant increase of *C. acnes*, accompanied by a decrease of *Staphylococcus epidermidis*, on the scalp of AA patients [81]. When studying microbiota profiling in diseases, one should also consider whether microbiome shifts represent a causative factor or simply an epiphenomenon secondary to the disease process.

Is the community function of microorganisms directly linked to their relative abundance? How diverse are metabolic pathways and networks within the follicular microenvironment? What effects does crosstalk between protein-coding genes and microbes have? Do environmental triggers affect the functioning and long-term stability of the HF? Now more than ever, technological advances in DNA collection and analysis could provide answers to such questions. Metagenomics represent a strategic concept in achieving a holistic view of the microbial world by investigating three interconnected levels—sample processing, DNA sequencing, and functional analysis [82].

## 6. A New Era of Microbiome Analyses

Metagenomics, the culture-independent genomic analysis of microbial DNA from environmental samples, allows the analysis of unculturable or previously unknown microbes [83]. Previous decades of research have proven how culturing is the perfect method to learn a lot about a tiny proportion of earth’s microorganisms [83]. Even though culturable bacteria represent less than 1% of the global bacterial species diversity [84], cultures have been the go-to diagnostic method for bacterial infections for decades [83]. Shotgun metagenomics is the non-targeted sequencing of all microbial genomes present in a sample. Combined with new sequencing technologies and computational pipelines, it has transformed recent microbiology research [85]. Even though profiling undescribed microbes is challenging, the reference-genomes produced yearly are quickly increasing (e.g., Human Microbiome Project, Human Microbiome Advanced Project), and accuracy improves as more high-quality metagenomic assemblies become available [85].

Metagenomics data allow taxonomic profiling (describing microbial species abundance) and subtyping of microorganisms according to their strain type [86]. There are two methods for strain level analysis: genotyping and phenotyping, but bacterial phenotypes cannot discriminate between closely related strains. Thus, subtyping of microorganisms shifted to genotyping, i.e., discriminating strains based on their genetic content [87]. Threats to human health underlie the need for strain level identification, e.g., increased virulence and transmissibility or antibiotic resistance of specific strains [88]. Different strains of the same species may have varying effects in the cellular properties of skin cells and were shown to induce variable immune responses to the host [89,90]. For example, different strains of the common human pathogens *S. aureus* and *Streptococcus pyogenes* induced acute adaptive immune response of great variability, partly explaining the clinical heterogeneity of infection from the same species among different patients [91].

Functional profiling and the characterization of the metabolic interactions of the microbiome with its human host constitute great potential advantages of sequencing, especially in the understanding of microbe-related diseases. Translated sequence searches against functionally characterized protein families can be used to interpret large metagenomic datasets, including the determination of gene families and pathways absent or present within a community [85,87]. Large metabolic pathway database projects such as the MetaCyc and KEGG, have been under development for years and provide reference pathways that are used to predict the metabolic pathways present in an organism from the annotated genome of that organism [92]. The Human Microbiome Project is a great example of an interdisciplinary world-wide effort to describe and understand the microbial components of human genetics and metabolic landscapes, as well as to elucidate their biological functional roles within their human environment, and how these contribute to physiology and disease [93]. Pipelines like HUMAnN have been further developed to determine the biological functions of these human bacterial communities, and reconstruct their metabolic activities [94].

As we try to identify the essential components of homeostasis and understand the dysfunctions of dysbiosis, biological mechanisms can be explored via novel computational methodologies and technologies that analyze the evolution of microbial communities and their genetic composition, microbe–host interactions, and the effects of external environmental triggers [95]. However, the implementation of these technologies in research or in the clinical and diagnostic routine is not without limitations. In addition to the high cost, there is a scarcity of personnel with the necessary computational training for the analysis of these complex metagenomic datasets. There is also still a lack of standardized computational tools in order to improve the reproducibility of these complex analytical methods.

## 7. Conclusions

It is not yet clear exactly what changes in the intra- and peri-follicular signaling milieu of alopecia patients, inciting alterations of normal HF- or skin immunology that eventually result in HF damage. It is still unknown whether the hair follicle’s IP collapse is an early stage of the disease pathogenetic process, or a secondary phenomenon as a result of environmental triggers such as dysbiosis. Answering these questions could help focus new therapeutic approaches toward the immune system or the HF unit itself. Various aspects of the microbiome have been correlated to human diseases. Furthermore, some microbiome-centric interventions have shown extraordinary efficacy in the treatment of specific disorders such as recurrent psoriasis, opening new avenues for therapeutic approaches to help reshape the human microbiome toward a healthy status. With this review, we hypothesize a possible connection between the HF microbiome and the pathogenesis of alopecia. We also show the potential of gaining novel knowledge on PCA pathogenesis by moving past culture-dependent approaches in an era of alternative tools, and by merging basic metagenomics approaches with new complementary technologies.

## Data Availability

Data sharing not applicable to this manuscript as no datasets were generated or analyzed for this work.
